# Exploring general practitioners' experience of informing women about prenatal screening tests for foetal abnormalities: A qualitative focus group study

**DOI:** 10.1186/1472-6963-8-114

**Published:** 2008-05-28

**Authors:** Cate Nagle, Sharon Lewis, Bettina Meiser, Jane Gunn, Jane Halliday, Robin Bell

**Affiliations:** 1 Murdoch Children's Research Institute, Royal Children's Hospital, VIC, Australia; 2 Department of General Practice, University of Melbourne, VIC, Australia; 3 Department of Paediatrics, University of Melbourne, VIC, Australia; 4 Prince of Wales Clinical School, University of New South Wales, NSW, Australia; 5 Women's Health Program, Department of Medicine, Monash University, VIC, Australia

## Abstract

**Background:**

Recent developments have made screening tests for foetal abnormalities available earlier in pregnancy and women have a range of testing options accessible to them. It is now recommended that all women, regardless of their age, are provided with information on prenatal screening tests. General Practitioners (GPs) are often the first health professionals a woman consults in pregnancy. As such, GPs are well positioned to inform women of the increasing range of prenatal screening tests available. The aim of this study was to explore GPs experience of informing women of prenatal genetic screening tests for foetal abnormality.

**Methods:**

A qualitative study consisting of four focus groups was conducted in metropolitan and rural Victoria, Australia. A discussion guide was used and the audio-taped transcripts were independently coded by two researchers using thematic analysis. Multiple coders and analysts and informant feedback were employed to reduce the potential for researcher bias and increase the validity of the findings.

**Results:**

Six themes were identified and classified as 'intrinsic' if they occurred within the context of the consultation or 'extrinsic' if they consisted of elements that impacted on the GP beyond the scope of the consultation. The three intrinsic themes were the way GPs explained the limitations of screening, the extent to which GPs provided information selectively and the time pressures at play. The three extrinsic factors were GPs' attitudes and values towards screening, the conflict they experienced in offering screening information and the sense of powerlessness within the screening test process and the health care system generally. Extrinsic themes reveal GPs' attitudes and values to screening and to disability, as well as raising questions about the fundamental premise of testing.

**Conclusion:**

The increasing availability and utilisation of screening tests, in particular first trimester tests, has expanded GPs' role in facilitating women's informed decision-making. Recognition of the importance of providing this complex information warrants longer consultations to respond to the time pressures that GPs experience. Understanding the intrinsic and extrinsic factors that impact on GPs may serve to shape educational resources to be more appropriate, relevant and supportive.

## Background

Recent developments have made screening tests for foetal abnormalities available earlier in pregnancy and women have a range of testing options accessible to them. It is now recommended that all women, regardless of their age, are provided with information on prenatal screening tests [1,2]. The provision of information is an essential component in facilitating women's informed decision-making [3-6]. At a minimum, women need to understand the condition(s) for which the testing is being offered, the characteristics of the test and the implications of testing [4].

Health professionals need to provide women with information on both the benefits and inherent limitations of screening tests in a way that optimises understanding, without increasing their level of anxiety [7-9]. This is particularly true of tests screening for foetal abnormality where decisions on diagnostic testing and/or termination of an affected pregnancy may arise.

The quality of information women have received has been found to be variable [4], while women have consistently demonstrated low levels of knowledge about prenatal screening for foetal abnormalities [3,5,6,10-13]. Consequently, there are challenges for all pregnancy care providers as well as implications for practice.

The introduction of screening tests in the first trimester of pregnancy has resulted in extending the role of General Practitioners (GPs). When providing confirmation of pregnancy, GPs are often the first health professional the woman consults. If this is in the first trimester, women are more likely to have all the prenatal test options available and have time to consider the information provided. As a primary health care provider the GP is well positioned to inform women of the increasing range of screening tests available.

Women value quality information that is provided by the health professional in a timely manner [14]. This requires the health professional to have a sound understanding of the relevant issues and be able to communicate this information. While studies have focused on exploring the knowledge and attitudes of health professionals towards prenatal testing [15-18], the effect on health professionals of providing this information to women has not received much attention. The purpose of this study was to explore GPs' experience of informing women about prenatal genetic screening tests for foetal abnormality.

## Methods

The study was conducted to inform the development of a decision aid for prenatal testing for foetal abnormalities, as part of a randomised controlled trial called the ADEPT (**A DE**cision aid for **P**renatal **T**esting) study [ISRCTN22532458] [19,20].

Focus groups interviews were used in preference to individual interviews as the synergistic effects of a group setting may result in the production of ideas that might not have been uncovered in individual interviews [21]. Purposive sampling [22] was employed to ensure both male and female GPs from rural and metropolitan practices participated, thereby increasing the heterogeneity of experiences and perspectives. Contact details were obtained from the on-line public telephone listings and GPs from targeted geographical areas were invited to participate in the study. GPs were also recruited from a variety of professional medical organisations. The team of investigators was comprised of a midwife (CN), an associate genetic counsellor (SL), a psychologist (BM), a general practitioner (JG) and two epidemiologists (JH and RB).

Informational redundancy was reached following four focus groups and no further groups were conducted in accordance with standard qualitative methodology [22]. The same facilitator (SL) and co-facilitator (CN) conducted all focus groups, using a semi-structured discussion guide to explore GPs' experiences. A literature review and the expertise of the project team informed the content of the question topics. The development of the discussion guide (Appendix) was informed by the method outlined by Greenwood and Parsons [23]. Audio-tapes of the sessions were professionally transcribed and the accuracy of the transcripts was verified using the raw data and field notes.

The analysis involved a constant comparison approach [24], with SL and CN repeatedly reading the transcripts to develop an agreed set of codes. The analysis was also guided by the conceptual qualitative research framework of Miles and Huberman [25]. One of the strengths of this framework lies in the explicitness of its description of qualitative data analysis, which includes the use of highly structured data displays to tease out relationships, synthesise findings and help guide against potential researcher bias. Data analyses were iterative and SL, RB & CN participated in this process to identify and agree upon emergent themes and discuss their face validity. The analysis took place concurrently with data collection, and results from each focus group were used to suggest additional lines of questioning in subsequent focus groups to ensure that divergent points of view would be expressed. The use of multiple coders and analysts and informant feedback are strategies recommended by Miles and Huberman to reduce the potential for researcher bias and increase the validity of the findings [25]. A draft manuscript was reviewed by four GPs who participated in different groups to authenticate the findings as representative of discussions and modify accordingly. In addition, a modified version of the clustered matrices described by Miles and Huberman [25] was used to facilitate analysis within and across groups. A code was used to de-identify responses comprising of a sequence of two digit codes for the group and the participant. Ethics approval was obtained from the Royal College of General Practitioners, Australia (ref NREEC 03-16) to conduct the study. All participants provided written consent to participate in the study.

## Results

Two groups were held in metropolitan locations and two in regional settings in Victoria, one of the south-eastern states of Australia, where approximately 63,000 women give birth each year [26]. In Australia the GP's role can vary from, at a minimum, confirming pregnancy and providing referral, to sharing pregnancy care with another provider (midwife, obstetrician) through to providing care in labour and the postnatal period. Prenatal care arrangements vary in different Australian States; in Victoria, women booked to give birth at publicly funded maternity services have access to a second trimester maternal serum screening test free of charge. Women in the public system who are at increased risk of foetal abnormality on the basis of their screening test result or their age (37 years or older by expected due date), are offered a funded diagnostic test at a tertiary hospital. Other screening tests, including nuchal translucency, combined first trimester screening and second trimester foetal anomaly ultrasounds, as well as diagnostic tests are available to all women on a fee for service basis.

In total twenty-seven GPs expressed interest and all participated: 18 female and 9 male. Six themes important in understanding GPs' experiences of informing women about screening tests were identified as common across the four groups. The themes were conceptualised as either intrinsic or extrinsic factors. Intrinsic factors describe the elements within the context of the consultation that impact on GPs. Extrinsic factors describe elements that operate beyond the scope of the consultation and have a broader impact on GPs. To further illustrate these six themes, the roles GPs adopt in response to both intrinsic and extrinsic factors are detailed in Table 1.

**Table 1:** Conceptual framework for understanding the impact of GPs' role in informing women of prenatal screening tests

**Factors**	**Role of the GP**
**Intrinsic Factors**	
1. Limitations of screening	Interpreter
2. Selectivity	Gatekeeper
3. Time pressures	Timekeeper
**Extrinsic Factors**	
4. Potential sequelae	Bearer of bad news
5. Conflict	Moral agent
6. Shifting sands	Intermediary

### Intrinsic Factors

Three factors occurring within the context of the consultation were consistently evident across all groups when the topic of screening was first broached by the GP. First, the limitations of screening was a central intrinsic factor. Another was the extent to which GPs filtered the complex information, and how they facilitated decision-making based on their own perceptions of the woman's information needs. The third related to time constraints, which was a pervasive factor across all aspects of communicating screening test information.

#### Limitations of screening

In order to inform women of screening tests, GPs related difficulties in communicating the complex information about the limitations of screening to women. Quotes to illustrate these views are shown in Table 2. All GPs reported presenting numerical expressions of probability in a way that was meaningful, particularly as there was strong agreement that women generally had a poor understanding of statistics.

**Table 2:** Limitations of screening: The GP as the interpreter

"You can't leave it and say "Well we'll just do the screening and then shall we think about should we have an amnio." I think you have to actually give people an idea of what to expect before you even embark on this whole thing." 0109
"I think you've got to tell them that the test is not 100% and that's where I think it gets complicated – and that's where you've got difficulties with people who can't understand statistics -I can't even understand it." 0201
"Bottom line is there are no guarantees for anything. " 0108
"We should be explaining the greyness...... there's almost nothing that we can offer and almost nothing we can do, will have an absolutely definite black or white answer." 0109
"It needs to be made clear that the result is not foolproof -it's not absolute." 0103
"It's very hard to get that message through until they get the gold standard answer." 0301

Communicating the inherent limitations of screening tests to the woman was reported as being a challenging experience for GPs. For many GPs, central to interpreting this complex information for women was the issue of grappling with the notions of risk and uncertainty, which challenged GPs on a number of levels. Not being able to provide a definitive answer was perceived as being a particularly difficult task. For some GPs, screening test discussions exposed their own lack of confidence or skill in dealing with screening test information. Providing a pre-test probability that was meaningful and conveying that a residual risk would exist, without causing anxiety, were also identified as difficulties.

#### Selectivity

GPs identified themselves as an important source of screening test information for the woman. There was considerable variation in the amount, level and delivery of information on screening tests that GPs provided. From the transcripts it was evident that many GPs provided information to women selectively, based on their perceptions of the woman's information needs, as well as the decision-making style they used within the consultation.

Acting as the 'gatekeeper of information', GPs demonstrated how testing can be presented differently to women, usually on the basis of maternal age or parity (Table 3). This was a deliberate process on the part of some GPs, yet for others it appeared to occur unintentionally. Some GPs who could be classified as providing information selectively to women also appeared to use a directive style within the consultation. GPs who indicated they communicated using a less directive decision-making style and involved the woman in decision-making, appeared less likely to act in a gatekeeper role.

**Table 3:** Selectivity: GP as the gatekeeper

"I certainly bring it up with everybody and give the various options that are available and I suppose I tend to push depending on what age that the mothers are." 0305
"If they're old I'll tell them I want you to think about it – if they're young, the subject is raised and then come back. And they often don't come back." 0204
"I think it's not good for doctors to assume just because someone's younger they wouldn't be interested or just because someone's old they would be interested, and to try and find the right language for that is quite challenging." 0404
"I still don't think women are going to understand what's best for them – they're going to rely on you to tell them what you think is best for them." 0203
"I think we can only guide the patients to the best decision that they are going to be comfortable with." 0304

When asked who tended to raise the issue of prenatal testing, seldom was the provision of screening information explained in terms of being at the woman's initiation. When women did raise the issue of testing, it was often explained in terms of women asking for the GP's personal recommendation, "...*that's a question I commonly get asked – what would I do?*" 0204

#### Time pressures

GPs described time pressures related to informing women about prenatal testing for foetal abnormalities as a significant source of stress. Time constraints were experienced because of the limitations of screening, the need for the information to be provided early in pregnancy and the competing pregnancy-related information the GP wanted to provide to the woman (quotes shown in Table 4). The first consultation in pregnancy was described as being information-laden and the discussions about testing increased time pressures.

**Table 4:** Time pressures: GP as the Time keeper

"I give them written information on the first visit but I usually stress out that...time will run out, so if they don't actually make up their mind reasonably quickly..." 0103
"I didn't used to bring up screening, but now because there's a bit of pressure...timing for the tests. I do I just say are you interested in screening?" 0202
"it's a lot to go through.... not to eat various foods, and you've got to tell them not to tell the world due to the risk of miscarriage, and you've got to tell them they can't have any alcohol and there's a lot to go through so it's fitting it all in." 0303
"To really discuss things in a meaningful way and give them information that they need to make a valid decision does become a fairly lengthy process." 0305
" it's very time-consuming to try and go through the issues when they first walk in..." 0404

The GP was seen as being required to act as a 'timekeeper' because screening test information was said to take a considerable amount of time to explain. Consistently GPs related that they had insufficient time to cover the topic and that it required an entire consultation, but seldom was this possible.

The introduction of first trimester screening tests has meant that it is often not appropriate for the GP to defer a discussion of these tests. GPs described how this imperative of time had negative consequences for the woman and themselves.

### Extrinsic Factors

The following three factors have been classified as extrinsic as they consist of elements that impact on the GP, beyond the scope of the consultation. They reveal GPs' attitudes and values to screening and to disability, as well as raising questions about the fundamental premise of testing. Extrinsic factors also include perceptions by GPs of their role within the screening test process and the health care system generally.

#### Potential sequelae

In providing information about screening tests to women, GPs voiced the importance of addressing why the tests were being offered and the possible consequences of having a screening test. GPs identified the negative impact on GPs of providing this information earlier in pregnancy than was required previously when women presented, happy with the confirmation of their pregnancy, and the GP needed to introduce the topic of testing (Table 5).

**Table 5:** Potential sequelae: GP as the bearer of bad news

"I found it quite a downer. I mean they're all so excited about being pregnant and then within minutes we're talking about let's do a test to find out if you might need to terminate this pregnancy." 0304
"A lot would come in, they're overjoyed to be pregnant... and the thought of them throwing in testing for all those things that might be wrong with the pregnancy. It's a bit of a downer." 0103
"...In fact you feel like a bit of a spoilsport bringing it up because they're really happy and you know- and then your sort of saying Oh well you know I hate to bring it up but, have you thought about..." 0405

The GP was cast in the role of the 'bearer of bad news' through introducing the topic of testing which included the risk of foetal abnormality, the limitations of screening, the role of diagnostic testing and the option of termination of pregnancy in the event of a major abnormality.

#### Conflict

The apparent moral obligation to provide information on screening tests was in conflict with the GPs' perceptions of screening tests as having potentially negative implications for the woman, society and themselves (Table 6). The inherent limitations of a population screening approach further exposed this conflict. In their role as the 'moral agent', providing information on testing and offering the tests was identified by some GPs as having the potential to do more harm than good.

**Table 6:** Conflict: The GP as a moral agent

"I find it quite hard to be scientific and disconnected about it and yet still also taking into account someone's you know, value system." 0404
"You know some of them actually have the idea that because you're testing for it you've got to do something to fix it and they don't realize that there isn't anything you can do but terminate or decide to go through with the pregnancy." 0107
"What is meaningful information ?- even what is spina bifida really? and what is Down syndrome?.. are you going to terminate someone who has a great personality and a reasonably happy life? There are lots of issues...promoting...the perfect life, the perfect child, the perfect outcome whether we should you know – all those kind of things." 0202
"The other trick with that though is the perception of some people that it's not a risk – that it's alright to have a baby with a disability and therefore you know, how do you quantify that it's a risk you know... so how do you identify someone like that and not you know um, stir them up or upset them. It's tricky." 0404

GPs related a concern for women who would be made unduly anxious about the possibility of foetal abnormality. This was particularly true for young women where "a very large number" of young women would be *"put through the anxiety" *(0303) of testing for a single woman to benefit.

GPs in the role of the 'moral agent' were also concerned with the difficulty of *"taking into account someone's own ethical or moral perspective"*(0404). Using language that did not bring a personal value system into the consultation was viewed as troublesome.

The provision of information to women on screening tests caused some GPs to question how disability was being valued in society and whether or not they were implicated in promoting the 'perfect child'.

#### Shifting sands

GPs expressed issues of powerlessness relating to how they viewed their role in the screening process (Table 7). In part this was related to a difficulty in accessing and keeping up-to-date with testing information. This was discussed in terms of the demands of their generalist workload, numerous recent developments in screening as well as being up-to-date with practical aspects like cost and the quality of local testing providers.

**Table 7:** Shifting sands: GP as the intermediary

"There's been such massive changes in the last few years it's been hard to keep up with." 0306
"I still have some trouble on keeping up with what the changes are..." 0106
"It's not all that long ago that you tested after 37.5 years as a due date and under that you didn't sort of offer it and then it all sort of changed and now the lawyers have marched in and we now have to offer it everybody." 0303
"There's many pieces of information, but we can't all have a handle on because the ground's shifting, the tests are changing fairly rapidly.." 0304

GPs in the role of the 'intermediary' described themselves as acting as an advocate for the women, trying to empower them to make an informed choice regarding testing and avoid being coerced into testing by obstetricians who may present testing as "routine" or "compulsory".

GPs also described the frustration of poor communication from other health care providers and from problems with the screening system particularly in offering screening tests to women who are "not fully informed."

## Discussion

The findings of our study expose the considerable impact that informing women of prenatal screening tests for foetal abnormality has on GPs. The identification of intrinsic and extrinsic factors present a framework of describing the observed differences between factors that are contained within the consultation and factors that have a more pervasive impact on the GP. The consistency of the findings across different focus groups underscores the salience of the experience of GPs described. We propose that in order to improve women's capacity to make an informed decision about screening, attention needs to be directed to better support the GP's role.

The impact on GPs that we have described is only partially explained by their lack of knowledge. In particular, responsive professional development activities need to be directed to the complexity of factors impacting on the GP experience. Educational activities that embrace intrinsic factors should include epidemiological content and focus on communicating complex issues like 'risk' and 'uncertainty'[27]. A framework such as the patient-centred clinical method [28] within an interactive learning environment could provide the opportunity for GPs to explore ways to 'find common ground' in decision-making and be less directive in the information they provide.

GPs already work within significant time constraints. Other health professionals have previously reported the intrinsic factor 'time pressures' experienced by GPs in our study when informing women of screening [29,30]. The number of options for prenatal testing and the availability of earlier tests make extra demands on the GP's already limited time. The lack of time available to discuss prenatal testing is at odds with the increasing importance of the GP's role in informing women of screening. With the utilisation of prenatal screening increasing [31], it is timely to consider how to increase the amount of time allocated to consultations in early pregnancy. One option applicable to the Australian health care system might be to fund these complex discussions as long consultations, which attract a higher Medicare (Australia's publicly-funded universal health care system) rebate.

Extrinsic factors that impact on the GP beyond the scope of the consultation also warrant consideration. Our findings indicate the importance of GPs' values and attitudes to tests, screening and disability, in explaining how they experience their role in informing women. It is important that educational activities acknowledge and embrace the range of values and attitudes that exist. It may also be beneficial to consider creating forums for peer discussions about challenging experiences.

Even in a group practice, GPs often function in an isolated manner with limited opportunity to discuss issues relating to consultations. For example, during one focus group a GP revealed a professional concern to the group that the GP had not talked about previously to anyone. In light of the difficulties experienced by GPs in this study, a mechanism for GPs to share and reflect on their experiences may prove beneficial. For instance, the experience of genetic counsellors promote the benefit of 'supervision' [32], supporting the professional and providing a non-threatening environment to discuss psychosocial aspects of consultations.

## Conclusion

The purpose of using a qualitative design to address this research question was to document the *range *of GP experiences to facilitate an in depth analysis of issues in the hope that these finding will make a valuable contribution to achieving a better understanding of GPs' experience of screening. However, only quantitative studies can unpack the *degree *to which these experiences and beliefs are endorsed.

This study provides a lens through which to view complex primary health consultations. Facilitating women's informed decision-making regarding prenatal screening tests requires much more from the GP than the provision of appropriate information under significant time constraints; moral dimensions also exert an influence.

An understanding of epidemiological language and communication skills is needed by GPs to assist them in providing women with meaningful information. However, it is important that the scope of future educational programs is broader than solely providing GPs with information and education. Consideration of GPs' values and attitudes may well serve to make educational resources more appropriate, relevant and supportive. Educational activities that fail to explore attitudes and values of participants to disability, screening and termination of pregnancy may be considered fundamentally flawed.

## Competing interests

The authors declare that they have no competing interests.

## Authors' contributions

CN, SL and RB generated the research question. BM provided expertise on methodology. SL and CN conducted the focus groups. CN, SL conducted the coding and analysis with supervision from and RB. JH and JG provided expertise in interpretation of the results. CN wrote the first draft and all other authors contributed to subsequent drafts and approved the final version.

## Appendix: discussion guide for focus groups

When I say the words "prenatal testing for Down syndrome", what is the first thing that comes into your mind?

Generally, how much do you think GPs know about prenatal testing?

How often does prenatal testing come up as a topic for discussion during your consultations with pregnant women?

Do you raise the issue of prenatal testing with pregnant women or do they tend to bring it up?

How do you go about discussing prenatal testing with pregnant women?

What questions do women commonly ask you about prenatal testing?

How much time do you spend in discussing prenatal testing with women?

Tell me about your recent experiences discussing prenatal testing with your patients.

What do you find most difficult about discussing prenatal testing?

What are the key points about prenatal testing that you think women need to understand in order to make a decision about what is best for them?

What resources do you use to discuss prenatal testing and how helpful are they?

Are there other resources that you use for other health related matters that you commonly use that you find useful?

When do you think would be the best time to discuss prenatal testing and hand out a decision aid?

Are there any other issues that you would like to raise?

## Pre-publication history

The pre-publication history for this paper can be accessed here:


